# MaNmrA, a Negative Transcription Regulator in Nitrogen Catabolite Repression Pathway, Contributes to Nutrient Utilization, Stress Resistance, and Virulence in Entomopathogenic Fungus *Metarhizium acridum*

**DOI:** 10.3390/biology10111167

**Published:** 2021-11-12

**Authors:** Chaochuang Li, Qipei Zhang, Yuxian Xia, Kai Jin

**Affiliations:** 1 Genetic Engineering Research Center, School of Life Sciences, Chongqing University, Chongqing 401331, China; leezc90@163.com (C.L.); zhangqipei12201@yeah.net (Q.Z.); 2 Chongqing Engineering Research Center for Fungal Insecticide, Chongqing 401331, China; 3 Key Laboratory of Gene Function and Regulation Technologies under Chongqing Municipal Education Commission, Chongqing 401331, China

**Keywords:** *Metarhizium acridum*, *MaNmrA*, nutrition utilization, stress response, virulence

## Abstract

**Simple Summary:**

Nutrient metabolism is closely related to the growth, development, and pathogenicity of pathogenic fungi. The nitrogen catabolite repression (NCR) pathway is a well-known fungal nitrogen source regulation path, in which NmrA plays an important regulatory role. Here, we reported a negative regulatory protein MaNmrA, the NmrA homologous protein, in the entomopathogenic fungus *Metarhizium acridum*, and found that it played important roles in carbon and nitrogen metabolism, growth, stress tolerance, and virulence of *M. acridum*. Our work will provide a theoretical basis for further exploring the functions of NCR pathway related genes in entomopathogenic fungi.

**Abstract:**

The NCR pathway plays an important regulatory role in the nitrogen metabolism of filamentous fungi. NmrA, a central negative regulatory protein in the NCR pathway and a key factor in sensing to the carbon metabolism, plays important roles in pathogenic fungal nutrition metabolism. In this study, we characterized the functions of *MaNmrA* in the insect pathogenic fungus *M. acridum*. Multiple sequence alignments found that the conserved domain (NAD/NADP binding domain) of MaNmrA was highly conservative with its homologues proteins. Deletion of *MaNmrA* improved the utilization of multiple carbon sources (such as glucose, mannose, sucrose, and trehalose) and non-preferred nitrogen sources (such as NaNO_3_ and urea), significantly delayed the conidial germination rate and reduced the conidial yield. The *MaNmrA*-disruption strain (Δ*MaNmrA*) significantly decreased tolerances to UV-B and heat-shock, and it also increased the sensitivity to the hypertonic substance sorbitol, oxygen stress substance H_2_O_2_, and cell wall destroyer calcofluor white, indicating that loss of *MaNmrA* affected cell wall integrity, tolerances to hypertonic and oxidative stress. Bioassays demonstrated that disruption of *MaNmrA* decreased the virulence in both topical inoculation and intrahemocoel injection tests. Further studies revealed that the appressorium formation, turgor pressure, and colonization in hemolymph were significantly reduced in the absence of *MaNmrA*. Our work will deepen the functional cognition of *MaNmrA* and make a contribution to the study of its homologous proteins.

## 1. Introduction

Entomopathogenic fungi are important insect pathogenic microbes and play important roles in the control of agricultural pests [1]. Among them, *Beauveria* spp. and *Metarhizium* spp. are the most widely used for the prevention of agricultural and forest pests [2]. Insect pathogenic fungi are the only kind of microbes that can directly penetrate host cuticle, while the bacteria and viruses generally infect the host through the oral cavity or wound. Conidia of entomopathogenic fungi firstly adhere to the host cuticle, then germinate to form infection structure appressoria, followed by penetrating the host cuticle under the action of turgor pressure and cuticle degrading enzymes, colonizing in the host hemolymph, and killing them [3,4]. For pathogenic bacteria, such as *Bacillus thuringiensis* (Bt), once Bt enters the host, it will produce different types of toxins or toxic proteins, which can destroy the host’s immune systems and ultimately lead to the death of the host [5,6]. In addition, insect pathogenic nematodes contain a large number of symbiotic bacteria in their intestines, which will be released and massively multiply in the host hemolymph, and eventually kill the host [5]. Conidia are the effective infection unit of pathogenic fungi, the activity, infection, and pathogenicity of the conidia are easily disturbed by the external environment, such as nutritional conditions, temperature, humidity and UV-B, etc. [7].

Nutrient elements play important roles in the growth and development of organisms. Nitrogen is an important component of a variety of biological macromolecules, such as proteins and nucleic acids, and occupies an important position in the life history of organisms. Generally, fungi can uptake a variety of nitrogen sources, such as nitrate, ammonium, and urea, etc., which involves multiple regulatory genes [8,9]. The most widely known in fungal nitrogen metabolism is the nitrogen catabolite repression (NCR) pathway, also known as the nitrogen metabolite repression (NMR) pathway, that is, fungi will preferentially assimilate the most preferred glutamine or ammonium, other nitrogen sources (such as nitrate, nitrite, and purine) can be utilized until the preferential nitrogen sources are consumed or in the absence of preferential nitrogen sources [7]. In filamentous fungi, the NCR pathway is mediated by the GATA transcription factors AreA and AreB, which are considered to have opposite biological functions in regulating nitrogen source utilization [10]. In the presence of the preferential nitrogen sources, NmrA interacts with the C-terminal of AreA to inhibit *AreA* activity and block the transcription of genes involved in assimilating other nitrogen sources, if not, NmrA will be separated from the NmrA-AreA heterodimer, restoring the activity of *AreA*, thereby promoting the expression of genes related to other nitrogen utilizations [11,12]. Under nitrogen starvation condition (with no preferential nitrogen sources), AreA mediates the derepression of genes involved in the utilization of non-preferential nitrogen sources [13], it also needs the cooperation of pathway specific transcription factor NirA with nitrate as the sole nitrogen source [14]. Furthermore, AreB and AreA play different roles in utilizing secondary nitrogen sources in different species. For example, AreB can negatively regulate nitrogen catabolism genes by competing with AreA for binding sites in *Aspergillus nidulans* [10,15] while *AreB* is activated by AreA and cooperates with AreA in response to nitrogen source changes in *Fusarium fujikuroi* [16].

NmrA, a central and negative regulator in the NCR pathway, can specifically bind to the cofactor dinucleotides NAD(P)^+^ and regulate the transcription of related genes by interacting with transcription factors [17,18]. Sufficient nitrogen source will promote the combination of NmrA with AreA or NIT2 (AreA homologous protein) to inhibit the expression of *AreA*/*NIT2* in *A. nidus* or *Neurospora crassa* [11,19], while the expression of *nmr1* (*NmrA* homologous gene) is strictly inhibited under sufficient nitrogen conditions in *F. fujikuroi* [20,21]. Furthermore, the bZIP transcription factor MeaB can specifically bind to *NmrA* and activate the expression of *nmrA* in *A. nidulans* [22]. However, another study showed that MeaB has slight or no effect on the transcription of *NmrA* [21]. Furthermore, NmrA is also involved in the growth and development, adaptability to adversity, pathogenicity, and carbon metabolism of the pathogenic fungi. For example, disruption of *nmrA* in *A. flavus* will increase the conidial yield and the number of microsclerotia, which is an important hypopus for filamentous fungi to enhance the adaptability to adverse environments, implying that NmrA also plays important roles in fungal conidiation and stress adaptability [23]. In addition, the *nmrA* mutant could not infect peanut seeds, suggesting that *nmrA* is an important regulator for the virulence of *A. flavus* [23]. However, deletion of *nmr* does not affect the virulence in *F. fujikuroi* [7,20]. Moreover, the proteins NmrA/Nmr1-3 are involved in the regulation of carbon catabolite repression (CCR) pathway in both *A. nidulans* and *M. oryzae* [24,25].

In conclusion, studies have shown that *NmrA* is an important functional gene in different species, thus, we suspect that the *NmrA* homologous gene *MaNmrA* may also have multiple functions in *M. acridum*. To this end, we cloned and characterized *MaNmrA* in *M. acridum*, it revealed that *MaNmrA* played important roles in regulating nutrition utilization, growth, and development of the conidia, stress tolerances and virulence of *M. acridum*. These data indicated the functional diversity of *MaNmrA* in the model insect pathogens *M. acridum*.

## 2. Materials and Methods

### 2.1. Strains and Culture Conditions

The *M. acridum* CQMa102 strain (wild-type, WT), *MaNmrA*-deletion strain (Δ*MaNmrA*), and complemented strain (∆*MaNmrA*::*MaNmrA*) were grown on quarter-strength saboraud dextrose yeast agar (¼SDAY: 10‰ dextrose, 2.5‰ peptone, 5‰ yeast extract, and 18‰ agar, *w*/*v*), Czapek-dox (CZA: 30‰ sucrose, 2‰ NaNO_3_, 1‰ K_2_HPO_4_, 0.5‰ MgSO_4_, 0.5‰ KCl, 0.01‰ FeSO_4_, and 18‰ agar, *w*/*v*) or modified CZA (with different nitrogen or carbon sources) at 28 °C. *Escherichia coli* DH5α competent cells (Solarbio, Beijing, China) were used for the vector construction. *Agrobacterium tumefaciens* AGL-1 competent cells (Solarbio, Beijing, China) were used for fungal genetic transformation.

### 2.2. Bioinformatics Analysis

All the protein sequences of NmrA homologues were downloaded from NCBI (https://www.ncbi.nlm.nih.gov/, accessed on 3 May 2019). NmrA protein domain was analyzed with SMART interface (http://smart.embl.de/, accessed on 3 May 2019). The physical and chemical properties of MaNmrA were analyzed with ExPASy (https://web.expasy.org/protparam/, accessed on 3 May 2019). DNAMAN program was used for multiple sequence alignment analysis. MEGA 7.0 was used for constructing the neighbor-joining tree under 1000 bootstrap replicates.

### 2.3. Creation of *MaNmrA* Mutants

The Δ*MaNmrA* and ∆*MaNmrA*::*MaNmrA* strains were constructed as described previously [26]. Briefly, the genome DNA of WT strain was used for amplifying the 5′ and 3′ flanking fragments of *MaNmrA* with primers NmrA-LF/NmrA-LR and NmrA-RF/NmrA-RR, followed by inserting into backbone vector to form the knockout vectors pK2-SM-*MaNmrA*-F and pK2-SM-*MaNmrA*-R, respectively (Figure S1A). The revertant fragment was amplified from the gDNA of WT strain with primers CP-F/CP-R and ligated into pK2-sur vector, forming complementation vector pK2-*MaNmrA*-sur (Figure S1B). All disruption and complementation vectors were transferred into AGL1 for the genetic transformation of *M. acridum* to obtain the Δ*MaNmrA* and ∆*MaNmrA*::*MaNmrA* transformants via the homologous recombination and random insertion principles. Putative mutants of the Δ*MaNmrA* and ∆*MaNmrA*::*MaNmrA* strains were screened with glufosinate ammonium (500 μg/mL) or chorimuron ethyl (20 μg/mL). The transformants were verified by PCR and further verified via Southern blotting (Figure S1C) with DIG High Prime DNA Labeling and Detection Starter Kit I (Roche, Basel, Switzerland). Primers used in this study are listed in Table S1.

### 2.4. Growth Characteristic Assays

To analyze the effects of *MaNmrA* on nitrogen and carbon utilization, the WT, Δ*MaNmrA*, and ∆*MaNmrA*::*MaNmrA* strains were grown on modified CZA supplemented with 25 mM glutamine (Gln), glutamate (Glu), (NH_4_)_2_SO_4_, NaNO_3_, and urea, or 88 mM glucose, fructose, galactose, mannose, sucrose, and trehalose, respectively. Two microliters conidial suspensions (10^6^ conidia/mL) of each strain were inoculated onto the modified CZA plates containing different nitrogen or carbon sources and incubated at 28 °C for 7 days. To detect the effects of *MaNmrA* on the conidial germination and hyphal growth, 100 μL conidial suspensions at a concentration of 10^7^ conidia/mL of each strain were spread on ¼SDAY media and incubated at 28 °C, followed by recording the conidial germination of each strain every 2 h and photographing the micro-morphological development characteristics of hyphae with a digital light microscope. To determine the conidial yield, 2 μL conidial suspensions (10^6^ conidia/mL) were inoculated onto the ¼SDAY solid media and then incubated at 28 °C for days to count the conidial yield [27]. Conidial suspensions (10^6^ or 10^7^ conidia/mL) of the WT, Δ*MaNmrA*, and ∆*MaNmrA*::*MaNmrA* strains were prepared with 0.05% Tween-80 after the fungal culturing for 15 days on ¼SDAY.

### 2.5. Stress Tolerance Analysis

To analyze the fungal sensitivities to different environmental stressors, 2 µL conidial suspensions (10^6^ conidia/mL) of each strain were respectively inoculated onto ¼SDAY plates with 0.05 mg/mL calcofluor white (CFW), 0.01% sodium dodecyl sulfate (SDS), 0.5 mg/mL congo red (CR), 6 mM H_2_O_2_, 1 M sorbitol, and 1 M NaCl, then cultured at 28 °C for 7 days (the plates containing CFW and H_2_O_2_ were incubated in the dark). The relative growth inhibition (RGI) was used to assess the inhibition of chemicals on the fungal strains. The tolerances of the fungal strain to UV-B and heat-shock were determined according to previous methods [28]. For the UV-B treatment, 50 μL 10^7^ conidia/mL conidial suspensions of each strain were spread on ¼SDAY plates and treated with 1350 mW/m^2^ UV-B for 1.25, 2.50, 3.75, or 5.00 h, which was provided by a 40-W fluorescent lamp with a total dose of 4.86 kJ/h·m^2^. For the heat-shock treatment, conidial suspensions (10^7^ conidia/mL) of the fungal strains were placed in sterile centrifuge tubes and dipped into a 45 °C water bath for 3, 6, 9, and 12 h, followed by pipetting and spreading 50 μL conidial suspensions on ¼SDAY plates, respectively. The germination rates of the treated strains were estimated with the 50% inhibition time (IT_50_) after incubating for 20 h.

### 2.6. Virulence Assays

To evaluate the effect of *MaNmrA* on the virulence, the bioassays were performed with fifth-instar nymph of *Locusta migratoria manilensis* through the methods of topical inoculation and intra-hemocoel injection in a previous study [29]. For topical inoculation, 5 µL conidial suspensions (10^7^ conidia/mL), prepared with paraffin oil, of the fungal strains were dropped on the pro-nota of the tested locusts, the locusts inoculated with 5 μL liquid paraffin oil served as the control. For intra-hemocoel injection, 5 µL conidial suspensions (10^6^ conidia/mL), prepared with sterile water, of the fungal strains were injected into the hemolymph of the tested locusts, the locusts injected with 5 μL sterile water served as the control. All tested locusts were fed in the bioassay room with a temperature of 28 °C, a photoperiod of 16 h:8 h (light:dark), and a relative humidity of 50–70%. The number of dead locusts was recorded every 12 h, and the virulence of the three strains was estimated with 50% lethality time (LT_50_). Each treatment (*n* = 30) was repeated three times.

To determine the growth of *M. acridum* in the locust hemolymph and the utilization of nutrition, 10 μL conidial suspensions (10^6^ conidia/mL) of the fungal strains were respectively added into 500 μL locust hemolymph, complete medium ¼SDY (¼SDAY without agar), or modified CZB (CZA without agar) with 88 mM trehalose as the single carbon source, then incubated in a shaker incubator at 28 °C with 220 rpm for two or three days, followed by collecting the fungal samples to quantify the gDNA concentration via qPCR with primers of the 18s rDNA ITS (internal transcribed spacer) sequence.

To analyze the development of infection structure appressorium, the conidial germination and appressorial formation of the fungal strains incubated on the locust hind wings were determined according to previously study [30]. Briefly, the locust hind wings were immersed in the conidial suspensions (10^7^ conidia/mL), prepared with 0.05% Tween-80, and placed on a tachometer and rotated at a low speed for 60 min. This was followed by taking out the wings, placing them on a clean glass slide and absorbing moisture, then the glasses were placed in a petri dish, which contained 5 pieces of filter paper evenly dripped with 2 mL ddH_2_O, followed by culturing at 28 °C for hours to count the conidial germination and appressorium formation. The appressorium collapsed was determined after treating with PEG8000 and the neutral lipids in the appressorium were determined after staining with Nile Red [30].

### 2.7. qRT-PCR Analysis

Appressoria of the fungal strains that were incubated for 24 h were used to determine the transcriptional level of genes involved in adhesion, cuticle-degrading, and glycerol-synthesis. Ultrapure RNA Kit (DNase Ⅰ) (CoWin Bio, Beijing, China), PrimeScript^TM^ RT reagent Kit with gDNA Eraser (TaKaRa, Dalian, China), and SYBR^®^ Premix Ex TaqTM (TaKaRa, Dalian, China) were used for extracting RNA, synthesizing cDNA, and qRT-PCR, respectively. The 2^−ΔΔCt^ method [31] was used for analyzing the data with an internal marker gene *gpdh* (EFY84384) in *M. acridum*.

### 2.8. Data Analysis

Microsoft Excel 2019 and SPSS 20.0 software were used for data processing. Graphpad Prism 8, Adobe Photoshop 2021, MEGA 7.0, and DNAMAN software were used for image processing. One-way ANOVA with Tukey’s HSD test was used for data (shown as the mean ± SD) analysis with significance level set at 0.05 or 0.01 using SPSS 20.0 software. All experiments were repeated more than three times.

## 3. Results

### 3.1. Identification and Sequence Features of NmrA Ortholog in M. acridum

**Figure 1:**
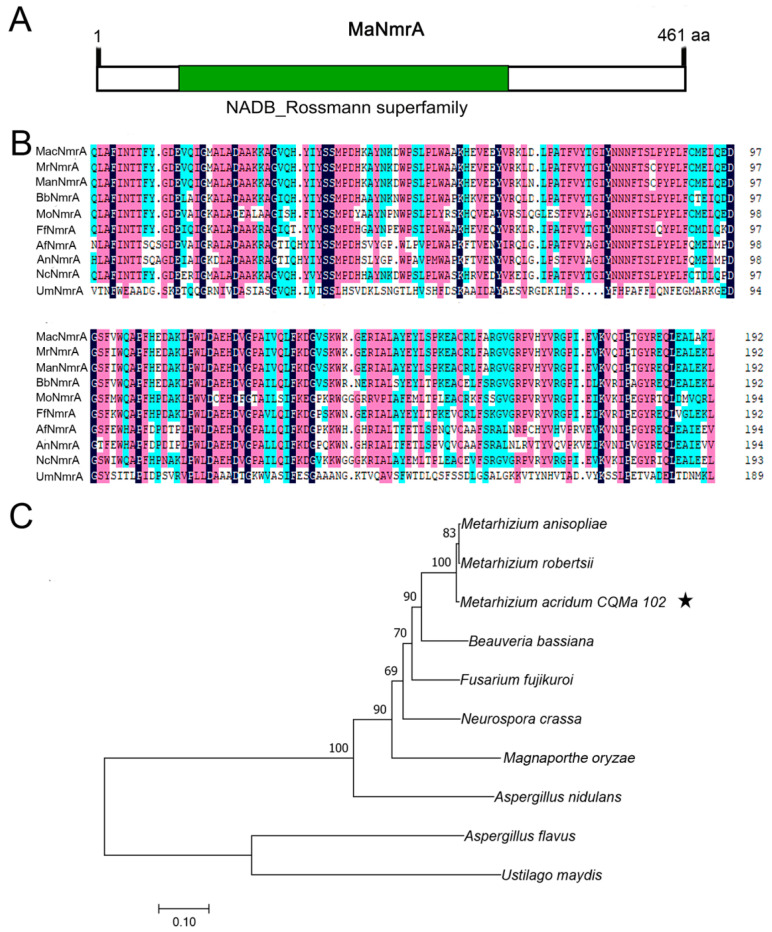
Conserved domain and phylogenetic analysis of MaNmrA. (**A**) Analysis of conserved domain in MaNmrA through SMART. (**B**) Multiple sequence alignments of the NAD or NADP binding domain among NmrA homologues. *Mac*, *Metarhizium acridum* (XP_007807089.1, MAC_00749). *Mr*, *Metarhizium robberstii* (XP_007825607.1). *Man*, *Metarhizium anisopliae* (KFG78248.1). *Bb*, *Beauveria bassiana* (XP_008599776.1). *Mo*, *Magnaporthe oryzae* (XP_003715776.1). *Ff*, *Fusarium fujikuroi* (CAA75863.1)*. Af*, *Aspergillus flavus* (XP_002382762.1). *An*, *Aspergillus nidulans* (AAC39442.1). *Nc*, *Neurospora crassa* (XP_961314.3). *Um*, *Ustilago maydis* (XP_011390347.1). (**C**) Phylogenetic tree analysis of MaNmrA and its homologous proteins. The pentastar indicated MaNmrA protein.

Based on the amino acid sequences of NmrA in *Aspergillus* strains, its homologous protein MaNmrA (NCBI accession No. MAC_00749) was retrieved in *M. acridum* through NCBI blastp alignment. The whole DNA sequence of *MaNmrA* was 1386 bp with no intron and MaNmrA protein contained 461 amino acids with an isoelectric point of 5.25 and a protein mass of 51.84 kDa. Further analysis in silico via SMART found that MaNmrA protein had a typical NAD or NADP binding domain with a core Rossmann type fold (Figure 1A). Multiple sequence alignments of the conserved domain (NAD or NADP binding domain) in NmrA homologues showed that MaNmrA was highly conservative with its homologues, and the identity was up to 93% (Figure 1B). The phylogenetic tree analysis revealed that MaNmrA was relatively close to entomopathogenic fungi *Metarhizium* and *Beauveria* (Figure 1C).

### 3.2. Deletion of *MaNmrA* Affected the Nitrogen and Carbon Utilization

**Figure 2:**
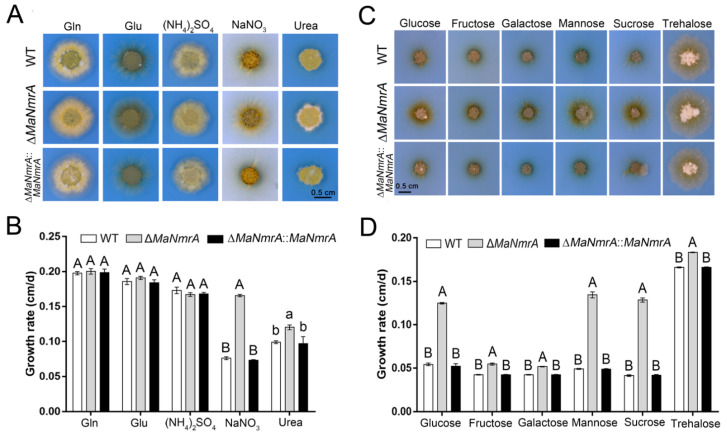
Deletion of *MaNmrA* affected the nitrogen and carbon utilization. Colony morphology (**A**) and growth rate (**B**) of the WT, Δ*MaNmrA* and ∆*MaNmrA*::*MaNmrA* strains on modified CZA medium supplemented with 25 mM Gln, Glu, (NH_4_)_2_SO_4_, NaNO_3_, and urea, respectively. Colony morphology (**C**) and growth rate (**D**) of the WT, Δ*MaNmrA*, and ∆*MaNmrA*::*MaNmrA* strains on modified CZA medium supplemented with 88 mM glucose, fructose, galactose, mannose, sucrose, and trehalose, respectively. Letters A, B, a and b above graph were used to shown the significant difference. a and b, *p* < 0.05. A and B, *p* < 0.01 (Tukey’s HSD).

To explore the function of the *MaNmrA* gene, the Δ*MaNmrA* and ∆*MaNmrA*::*MaNmrA* strains were obtained according to principles of homologous recombination and random insertion, respectively (Figure S1). Based on the important regulatory role of NmrA in the NCR pathway, we firstly focus on the role of *MaNmrA* in nitrogen utilization. The WT, Δ*MaNmrA*, and ∆*MaNmrA*::*MaNmrA* strains were inoculated onto the modified CZA medium with Gln, Glu, (NH_4_)_2_SO_4_, NaNO_3_, and urea as the sole nitrogen source, respectively. The results showed that the colonies of the Δ*MaNmrA* strain were larger than that of the WT and ∆*MaNmrA*::*MaNmrA* strains (Figure 2A), the average growth rates were significantly accelerated (Figure 2B), suggesting that disruption of *MaNmrA* significantly improved the utilization of non-preferential sources (such as nitrate and urea) of *M. acridum*.

Previous studies have reported that NmrA or its homologous proteins are involved in the CCR pathway, which play important roles in regulating the carbon source utilization of fungi. To investigate whether *MaNmrA* also affected carbon source utilization, all these strains were inoculated onto the modified CZA plates with glucose, fructose, galactose, mannose, sucrose, and trehalose, respectively. It showed that the hyphae of the Δ*MaNmrA* strain were more developed on all tested carbon source media (Figure 2C), and the average growth rates were significantly accelerated compared to the WT and ∆*MaNmrA*::*MaNmrA* strains (Figure 2D). These results indicated that loss of *MaNmrA* affected the utilization ability of multiple carbon sources in *M. acridum*.

### 3.3. Disruption of *MaNmrA* Affected Conidial Germination and Conidial Yield

**Figure 3:**
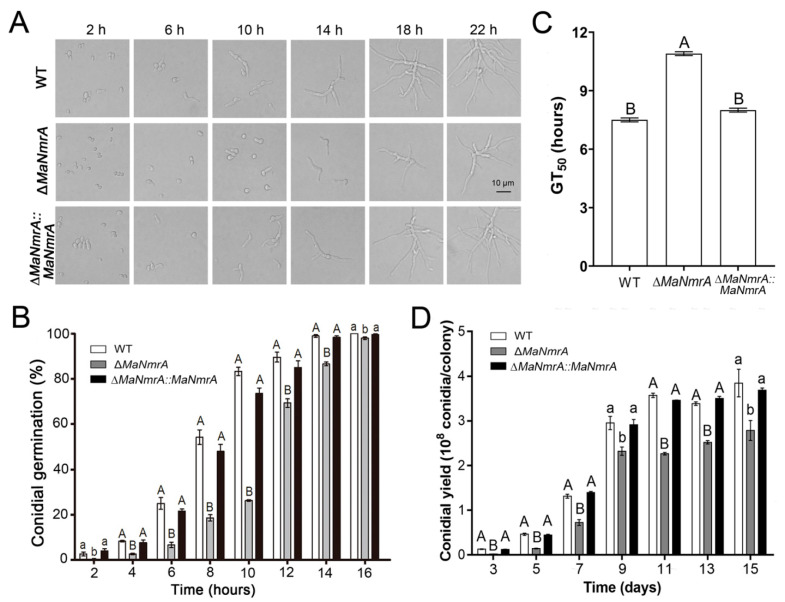
Disruption of *MaNmrA* delayed conidial germination and reduced conidia production. Growth (**A**), conidial germination rates (**B**), GT_50_s (**C**), and conidial yield (**D**) of the WT, Δ*MaNmrA*, and ∆*MaNmrA*::*MaNmrA* strains grown on ¼SDAY media at 28 ℃ for different hours or days. Letters A, B, a and b above graph were used to shown the significant difference. a and b, *p* < 0.05. A and B, *p* < 0.01 (Tukey’s HSD).

To clarify the effect of *MaNmrA* gene on the conidial growth and development, we determined the conidial growth characteristics of the fungal strains grown on ¼SDAY plates. It can be seen intuitively that the conidia of the WT and ∆*MaNmrA*::*MaNmrA* strains began to germinate after culturing for 2 h, while the conidia of the Δ*MaNmrA* strain had not yet germinated, which only had a few germinating conidia even cultured for 6 h. Furthermore, the conidial production of the Δ*MaNmrA* strain was obviously decreased compared to the WT and ∆*MaNmrA*::*MaNmrA* strains, which began to yield conidia after culturing for 18 h (Figure 3A). The germination rates of the Δ*MaNmrA* mutant at all tested time points were significantly delayed compared to that of the WT or ∆*MaNmrA*::*MaNmrA* strain (Figure 3B), and the half germination time (GT_50_) of the Δ*MaNmrA* strain (10.94 ± 0.08 h) was significantly increased compared to the WT (7.53 ± 0.16 h) or ∆*MaNmrA*::*MaNmrA* (8.01 ± 0.14 h) strain (Figure 3C). In addition, the conidial yield was significantly decreased in the absence of *MaNmrA* (Figure 3D). Taken together, these data indicated that *MaNmrA* play important roles in regulating the conidial germination, growth, and conidiation of *M. acridum*.

### 3.4. Disruption of *MaNmrA* Affected the Fungal Stress Tolerances

**Figure 4:**
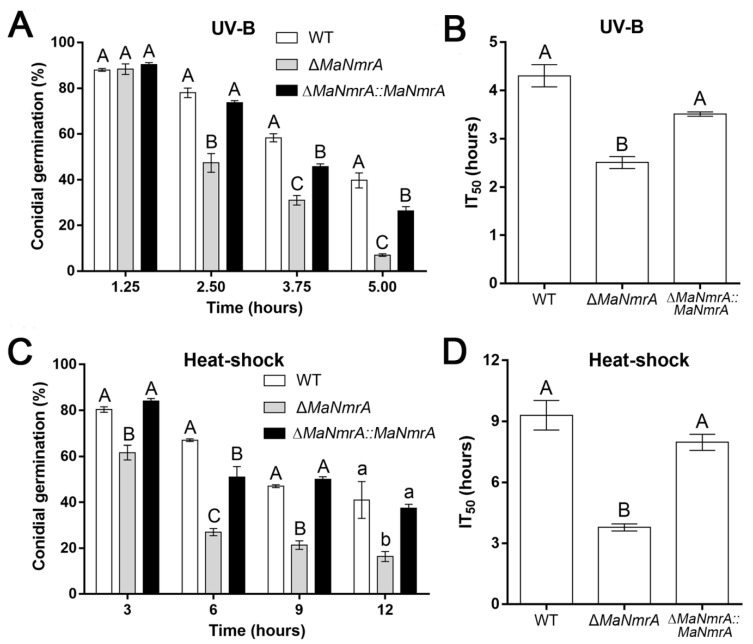
Disruption of *MaNmrA* reduced the tolerances to UV-B and heat-shock. Conidial germination (**A**) and the IT_50_s (**B**) of the WT, Δ*MaNmrA*, and ∆*MaNmrA*::*MaNmrA* strains after treating with UV-B. Conidial germination (**C**) and the IT_50_s (**D**) of the fungal strains after treating with heat-shock. Letters A, B, a and b above graph were used to shown the significant difference. a and b, *p* < 0.05. A and B, *p* < 0.01 (Tukey’s HSD).

To explore the response to stress conditions of the *MaNmrA* gene, we determined the tolerances to UV-B irradiation and heat-shock of the WT, Δ*MaNmrA*, and ∆*MaNmrA*::*MaNmrA* strains. After treating with UV-B, it was obviously found that conidial germination rate of the Δ*MaNmrA* strain was significantly reduced after 2.50, 3.75, and 5.00 h of treatment (Figure 4A), the half inhibition time (IT_50_) of the Δ*MaNmrA* strain (2.51 ± 0.18 h) was decreased compared to the WT strain (4.31 ± 0.33 h) and ∆*MaNmrA*::*MaNmrA* strain (3.51 ± 0.07 h) (Figure 4B). After treating with heat-shock, the conidial germination rate of the Δ*MaNmrA* strain was significantly reduced at all tested time points (Figure 4C), the IT_50_ of the Δ*MaNmrA* strain (3.78 ± 0.24 h) was significantly lower than that of the WT strain (9.30 ± 1.03 h) or ∆*MaNmrA*::*MaNmrA* strain (7.97 ± 0.55 h) (Figure 4D). These results showed that the tolerances to UV-B and heat-shock were significantly weakened in the absence of *MaNmrA*. It suggested that *MaNmrA* played important roles in the resistances to UV-B and heat-shock stress of *M. acridum.*

**Figure 5:**
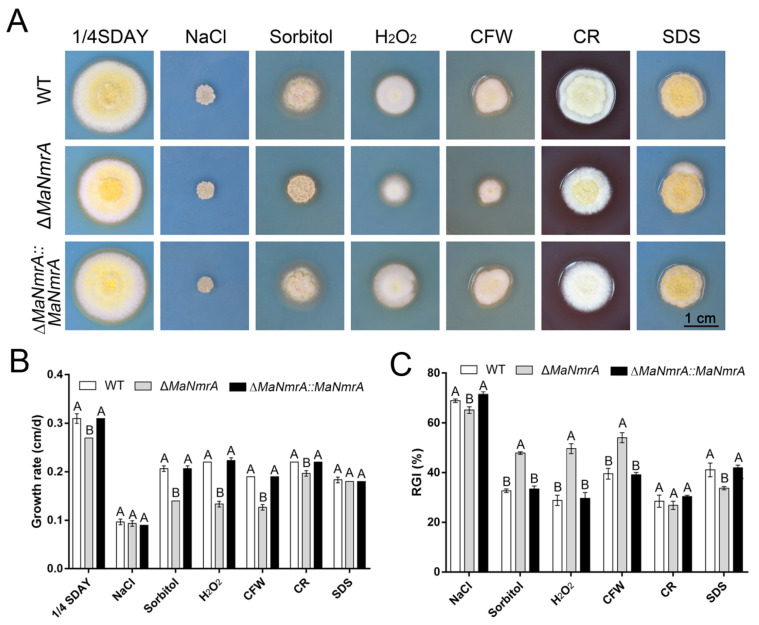
Disruption of *MaNmrA* reduced tolerances to multiple chemical reagents. (**A**) Colony morphology of the WT, Δ*MaNmrA*, and ∆*MaNmrA*::*MaNmrA* strains grown on ¼SDAY plates supplemented with 1M NaCl, 1M Sorbitol, 6 mM H_2_O_2_, 0.05 mg/mL CFW, 0.5 mg/mL CR, or 0.01% SDS, respectively. The growth rate (**B**) and relative growth inhibition rate (RGI) (**C**) of the fungal strains grown on ¼SDAY with different chemical reagents. Letters A and B above graph indicate significant differences (*p* < 0.01, Tukey’s HSD).

To analyze the effect of *MaNmrA* on the cell wall integrity and its role in high salinity, hypertonicity, and other adversities of *M. acridum*, corresponding chemical reagents were respectively added into the ¼SDAY media to observe the growth of WT, Δ*MaNmrA*, and ∆*MaNmrA*::*MaNmrA* strains. The results showed that the Δ*MaNmrA* strain grew slowly on the ¼SDAY medium (Figure 5A,B). Although there was no difference in colony morphology of the Δ*MaNmrA* strain from that of WT and ∆*MaNmrA*::*MaNmrA* strains when grown on the ¼SDAY with NaCl or SDS (Figure 5A,B), the relative growth inhibition (RGI) analysis found that the sensitivity of Δ*MaNmrA* strain to NaCl and SDS was decreased (Figure 5C). In addition, the growth of Δ*MaNmrA* strain was decelerated when cultured on the plate added with the hypertonic substance sorbitol, oxygen stress substance H_2_O_2_, or cell wall destroyer CFW (Figure 5A,B), and the sensitivity of the Δ*MaNmrA* strain to these three chemical reagents was significantly increased (Figure 5C). These data indicated that loss of *MaNmrA* affected cell wall integrity, tolerances to hypertonic and oxidative stress of *M. acridum*.

### 3.5. Deletion of *MaNmrA* Decreased Virulence

**Figure 6:**
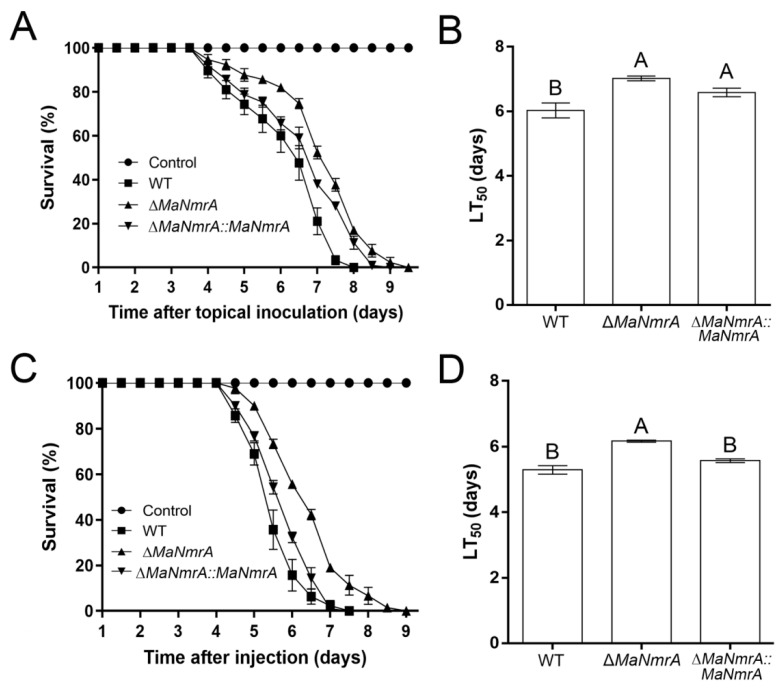
Disruption of *MaNmrA* decreased the virulence of *M. acridum.* (**A**) Survival of locusts respectively infected with WT, Δ*MaNmrA*, or ∆*MaNmrA::MaNmrA* strain via topical inoculation. (**B**) The LT_50_s of WT, Δ*MaNmrA* and ∆*MaNmrA*::*MaNmrA* strains in topical inoculation. (**C**) Survival of locusts respectively infected with WT, Δ*MaNmrA* or ∆*MaNmrA*::*MaNmrA* strain via intra-hemocoel injection. (**D**) The LT_50_s of WT, Δ*MaNmrA* and ∆*MaNmrA*::*MaNmrA* strains in intra-hemocoel injection. Letters A and B above graph indicate significant differences (*p* < 0.01, Tukey’s HSD).

To investigate the effect of *MaNmrA* to the pathogenicity of *M. acridum*, the bioassays were performed via the methods of topical inoculation and intra-hemocoel injection. It showed that the virulence of the Δ*MaNmrA* strain was significantly decreased in both these two tests. In topical inoculation test, the locusts infected with WT, Δ*MaNmrA*, and ∆*MaNmrA*::*MaNmrA* strains all died at 8, 9, or 9.5 dpi (days post inoculation), respectively (Figure 6A). The half lethality time (LT_50_) of the Δ*MaNmrA* strain (7.02 ± 0.11 d) was significantly delayed compared to the WT strain (6.03 ± 0.33 d) (Figure 6B). In intra-hemocoel injection test, locusts infected with WT, Δ*MaNmrA*, and ∆*MaNmrA*::*MaNmrA* strains were died at 7.5, 9.5, or 7.5 dpi, respectively (Figure 6C), LT_50_ of the Δ*MaNmrA* strain (6.17 ± 0.05 d) was significantly longer than that of the WT strain (5.29 ± 0.18 d) or ∆*MaNmrA*::*MaNmrA* strain (5.57 ± 0.08 d) (Figure 6D). These data showed that the pathogenic ability of *M. acridum* was decreased in the absence of *MaNmrA*.

**Figure 7:**
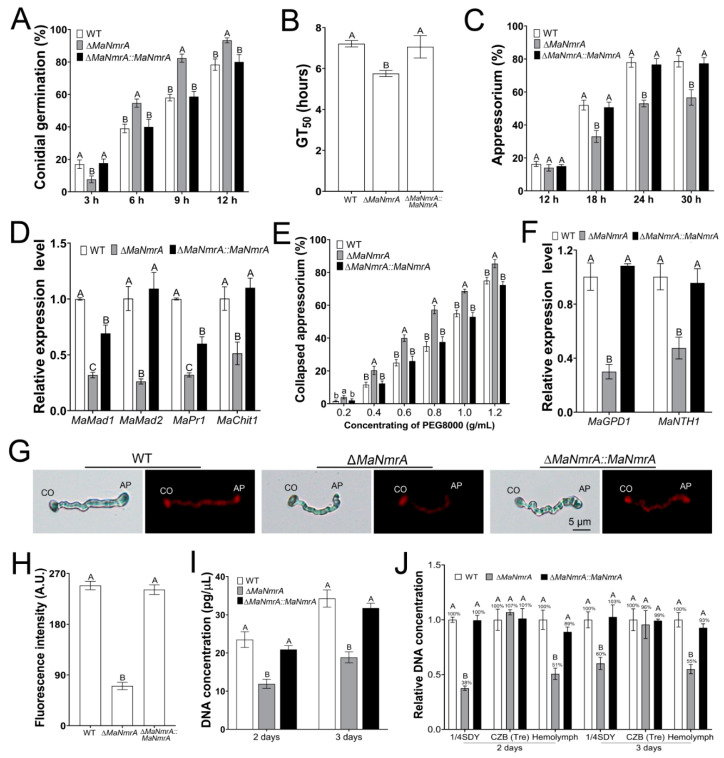
Deletion of *MaNmrA* affected appressorium development and the growth of the hyphal bodies. The conidial germination (**A**), GT_50_s (**B**), and appressorium formation (**C**) of the WT, Δ*MaNmrA*, and ∆*MaNmrA*::*MaNmrA* strains cultured on locust hind wings at 28℃ for different hours. (**D**) qRT-PCR analysis of genes involved in adhesion and cuticle-degrading. (**E**) The appressorium collapsed rates analysis. (**F**) qRT-PCR analysis of genes involved in glycerol synthesis. (**G**) Observation of lipid staining with Nile red. CO, conidium. AP, appressorium. (**H**) Fluorescence intensity measured in (**G**) through ImageJ software. A.U., Arbitrary Units. (**I**) Determination of the DNA concentrations of the fungal strains cultured in the hemolymph of locusts for 2 and 3 days. (**J**) Determination of the DNA concentrations of the fungal strains respectively cultured in complete medium ¼SDY, modified CZB (Tre), and locust hemolymph. CZB (Tre), CZB with 88 mM trehalose as the single carbon source. Fungal samples used for qRT-PCR analysis were incubated on locust hind wings for 24 h. Letters A, B, a and b above graph were used to shown the significant difference. a and b, *p* < 0.05. A, B and C, *p* < 0.01 (Tukey’s HSD).

Obviously, it showed that *MaNmrA* affected virulence by affecting the cuticle penetration process. It is known that appressoria play important roles in penetrating host cuticle [32], to this end, we tested the indicators related to the development of the appressoria. The germination rate of the Δ*MaNmrA* strain was significantly increased after incubating for 6, 9, and 12 h (Figure 7A), compared with the GT_50_ values of WT strain (7.21 ± 0.16 h) and ∆*MaNmrA*::*MaNmrA* strain (7.06 ± 0.55 h), the Δ*MaNmrA* strain had a lower GT_50_ (5.75 ± 0.15 h) (Figure 7B), however, the Δ*MaNmrA* strain formed fewer appressoria (Figure 7C). Furthermore, the expression levels of adhesion genes, *MaMad1* and *MaMad2*, and cuticle-degrading genes, *MaPr1* and *MaChit1*, were significantly decreased in the absence of *MaNmrA* (Figure 7D). To detect the appressorial turgor pressure, the appressoria were treated with different concentrations of PEG8000 and it showed that the collapsed rates were significantly increased at all tested concentrations in the absence of *MaNmrA* (Figure 7E). Moreover, the expression levels of glycerol-synthesis genes *MaGPD1* and *MaNTH1* were significantly decreased in the absence of *MaNmrA* (Figure 7F), and the fluorescence intensity of lipids was also decreased in the Δ*MaNmrA* strain (Figure 7G,H).

The virulence of Δ*MaNmrA* strain was also decreased in the intra-hemocoel injection test, suggesting that *MaNmrA* affected the colonization of *M. acridum* in locust hemolymph. To this end, we determined the growth of the fungal strains in the locust hemolymph and found that the genome DNA concentrations of the Δ*MaNmrA* strain, cultured in the hemolymph of locusts, were both significantly decreased after incubating for 2 and 3 days (Figure 7I). It revealed that the growth of the hyphal bodies was decreased in the absence of *MaNmrA*. To further explore the nutrient utilization of the Δ*MaNmrA* strain in host hemolymph, the WT, Δ*MaNmrA*, and ∆*MaNmrA*::*MaNmrA* strains were respectively inoculated in the locust hemolymph, complete medium ¼SDY, and modified CZB with the single carbon source trehalose, which is the blood sugar of insects and the sugar with the largest proportion in the hemolymph [33,34], then used quantitatively for the DNA content through qPCR. The results showed that the DNA concentration of Δ*MaNmrA* strain was significantly decreased under ¼SDY or hemolymph condition, and with no difference in CZB condition compared to the WT or ∆*MaNmrA*::*MaNmrA* strain when trehalose was the single carbon source (Figure 7J). It indicated that the growth of Δ*MaNmrA* strain in locust hemolymph may be related to the nitrogen source but not the carbon source.

## 4. Discussion

NmrA, a core regulator in the NCR pathway and containing the NADP binding site and NADB-Rossmann superfamily domains, can bind to NAD^+^/NADP^+^ [35] and is a conservative transcriptional regulatory factor that regulates the expression of related genes by interacting with transcription factor(s) [19]. In this study, we found that the conserved domain (NAD or NADP binding domain) of *MaNmrA* was highly conserved with that in other species. Subsequently, we obtained the Δ*MaNmrA* and ∆*MaNmrA*::*MaNmrA* strains to characterize the functions of *MaNmrA* and found that it had a multifunctional role in *M. acridum*.

Nitrogen metabolism is closely related to the growth and development of filamentous fungi. NmrA binds to the highly conserved C-terminal of GATA transcription factor AreA to negatively regulate the activity of *AreA* in the presence of a preferential nitrogen source, while NmrA is separated from the NmrA-AreA dimer to activate the expression of *AreA* and other genes involved in nitrogen catabolism and release the inhibition of nitrogen metabolism under nitrogen starvation condition [23,36,37]. In *A. flavus*, under the culture conditions with glutamine, ammonium, or proline as the nitrogen source, the colony edge of the Δ*NmrA* strain is more irregular compared with under other nitrogen sources [23]. In addition, previous studies have shown that the ability of AreA and AreB to sense the carbon metabolism is likely to depend on NmrA rather than on the transcription factor CreA, the core gene in the CCR pathway, under different carbon source conditions [25]. In *M. acridum*, the Δ*MaNmrA* strain could uptake nitrogen sources normally, the aerial hyphae of the Δ*MaNmrA* strain were increased when cultured on non-preferred nitrogen source conditions. Furthermore, the ability of Δ*MaNmrA* strain to utilize carbon sources was also significantly increased. The results showed that *MaNmrA* not only involved in nitrogen metabolism, but also played an important role in carbon metabolism.

In *A. flavus*, the conidial yield of Δ*NmrA* strain grown on PDA medium is no different from that of the WT strain, however, the conidial yield of Δ*NmrA* strain is significantly increased and the transcription levels of related regulatory genes are upregulated when ammonium is the sole nitrogen source [23]. In addition, deletion of *NmrA* inhibits the growth of *A. flavus* and increases the conidia production and microsclerotia significantly [23]. The microsclerotium is an important dormant body for filamentous fungi to enhance adversity adaptability, it implies that NmrA has important regulating effects on pathogenic fungi infection and environmental adaptability. In *M. acridum*, the Δ*MaNmrA* strain had a slower colony growth rate and a lower conidial yield compared to the WT or ∆*MaNmrA*::*MaNmrA* strain, indicating that *MaNmrA* may be involved in regulating hyphal growth and asexual sporulation.

The conidial size, germination rate, and adhesion on the host cuticle of entomopathogenic fungi are closely related to their pathogenicity [38,39,40,41]. High temperature and ultraviolet irradiation will weaken the activity of conidia and their ability to infect insect cuticle [42]. In addition, as an endophytic fungus, the colonization ability of *Metarhizium* in plant tissues is also related to environmental conditions, such as UV-B, temperature, and humidity [43]. Adapting to oxygen stress, osmotic pressure, and other complex environmental challenges and evading the host immune response are of great significance to the biocontrol fungi, such as *M. acridum*. Generally, pathogenic fungi will respond to the stress environments by regulating related regulatory factors or pathways [44,45], but some pathogenic fungi also produce some secondary metabolites to help improve their survivability in a stressful environment [46]. Once a pathogenic fungus enters the host hemocoel, it will induce the host to produce a series of immune responses, for example, a high osmotic pressure environment would be formed in host hemolymph to inhibit the reproduction of the microorganisms [32]. In this study, deletion of *MaNmrA* reduced the tolerances to UV-B and heat-shock, and significantly increased the sensitivity to hypertonicity, oxidants, and cell wall disruptors. In *A. flavus*, however, the *NmrA* deletion mutant has no effects on the hypertonic tolerance [23]. In summary, these results indicated that *MaNmrA* played important roles in the adaptability to adversity stress, suggesting that it may affect the pathogenicity of *M. acridum*.

Previous studies have shown that *NmrA* is necessary for the virulence of *A. flavus* [23]. Here, we confirmed that the virulence of Δ*MaNmrA* strain was reduced in both topical inoculation and intra-hemocoel injection tests. Further study found that the conidial germination rate of Δ*MaNmrA* strain was accelerated when culturing on the locust hind wings but was significantly decelerated when growing on the complete medium (¼SDAY). In general, due to the limitation of free moisture, conidia germinate slowly on the host cuticle [47]. However, the result was opposite in our work, and we speculate that it may be related to the dormancy of mature conidia, which will be affected by a variety of factors, such as blocked germination, senescence, or low intracellular water content [48]. Appressorium play important roles in the penetration process of entomopathogenic fungi, which could be enhanced by mechanical pressure provided by turgor pressure [32]. Here, we found that appressorium formation and turgor pressure were decreased in the Δ*MaNmrA* strain, indicating that *MaNmrA* seriously affected the cuticle penetration process. In addition, studies have shown that nitrogen source is a vital factor in conidial germination and appressorium formation of *B. bassiana*, the conidia cannot form germ tubes under the condition of nitrogen deficiency [49]. In *Metarhizium*, the level of nitrogen source directly determines appressorium formation [50]. Furthermore, the injection test showed that *MaNmrA* was also involved in the process of colonization in host hemolymph, the growth of *M. acridum* in host hemolymph was inhibited in the absence of *MaNmrA*. Meanwhile, we confirmed that *MaNmrA* did not affect the utilization of trehalose (insect blood sugar), indicating that it may be related to the utilization of nitrogen sources.

## 5. Conclusions

Nitrogen metabolism of most fungi, such as *Saccharomyces cerevisiae*, *A. nidulans*, *N. crassa*, and *M. oryzae*, is mainly regulated by the NCR pathway that is mediated by the GATA transcription factor AreA, which have been studied in multiple species. It is widely known that the auxiliary inhibitor NmrA is also a central member in the NCR pathway, it could initiate nitrogen repression or derepression by interacting with AreA or not. In this study, we characterized the functions of *MaNmrA* in the insect pathogenic fungus *M. acridum*. Deletion of *MaNmrA* improved the utilization of carbon and nitrogen sources, delayed the conidial germination, reduced the conidial yield, stress resistance, and virulence. In summary, these data provide a theoretical basis for further elucidating the mechanism of the NCR pathway influencing the growth, development, infection, and pathogenesis of insect pathogenic fungi.

## Data Availability

Not applicable.

## Supplementary Materials

The following are available online at https://www.mdpi.com/article/10.3390/biology10111167/s1, Figure S1: Vector construction and Southern blotting verification. Schematic diagram of *MaNmrA* knockout (A) and complemented (B) vector constructions. (C) Verification of the WT, Δ*MaNmrA* and ∆*MaNmrA*::*MaNmrA* strains by Southern blotting. Probe was amplified with primers NmrA-PF/NmrA-PR (Table S1). Restriction enzyme *APa*I was used to digest the gDNA of the fungal strains. Table S1: Primers used in this study.

## References

[1] Hyde K.D., Xu J., Rapior S., Jeewon R., Lumyong S., Niego A.G.T., Abeywickrama P.D., Aluthmuhandiram J.V.S., Brahamanage R.S., Brooks S. (2019). The amazing potential of fungi: 50 ways we can exploit fungi industrially. Fungal Divers..

[2] Faria M., Wraight S.P. (2007). Mycoinsecticides and Mycoacaricides: A comprehensive list with worldwide coverage and international classification of formulation types. Biol. Control..

[3] Goettel M.S., St Leger R.J., Rizzo N.W., Staples R.C., Roberts D.W. (1989). Ultrastructural localization of a cuticledegrading protease produced by the entomopathogenic fungus *Metarhizium anisopliae* during penetration of host (*Manduca sexta*) cuticle. Microbiology.

[4] Vega F.E., Meyling N.V., Luangsa-ard J.J., Blackwell M., Vega F., Kaya H.K. (2012). Fungal entomopathogens. Insect Pathology.

[5] Brivio M.F., Mastore M. (2020). When appearance misleads: The role of the entomopathogen surface in the relationship with its host. Insects.

[6] Pinos D., Andrés-Garrido A., Ferré J., Hernández-Martínez P. (2021). Response mechanisms of invertebrates to *Bacillus thuringiensis* and its pesticidal proteins. Microbiol. Mol. Biol. Rev..

[7] Tudzynski B. (2014). Nitrogen regulation of fungal secondary metabolism in fungi. Front. Microbiol..

[8] Caddick M.X. (1994). Nitrogen metabolite repression. Prog. Ind. Microbiol..

[9] Marzluf G.A. (1997). Genetic regulation of nitrogen metabolism in fungi. Mierobiol. Mol. Biol. Rev..

[10] Wong K.H., Hynes M.J., Todd R.B., Davis M.A. (2009). Deletion and overexpression of the *Aspergillus nidulans* GATA factor AreB reveals unexpected pleiotropy. Microbiology.

[11] Andrianopoulos A., Kourambas S., Sharp J.A., Davis M.A., Hynes M.J. (1998). Characterization of the *Aspergillus nidulans* nmrA gene involved in nitrogen metabolite repression. J. Bact..

[12] Wilson R.A., Arst H.N. (1998). Mutational analysis of AREA, a transcriptional activator mediating nitrogen metabolite repression in *Aspergillus nidulans* and a member of the ‘‘streetwise’’ GATA family of transcription factors. Microbiol. Mol. Biol. Rev..

[13] Wiemann P., Tudzynski B., Brown D.W., Proctor R.H. (2013). The nitrogen regulation network and its impact on secondary metabolism and pathogenicity. Fusarium: Genomics, Molecular and Cellular Biology.

[14] Narendja F., Goller S.P., Wolschuk M., Strauss J. (2002). Nitrate and the GATA factor AreA are necessary for in vivo binding of NirA, the pathway-specific transcriptional activator of *Aspergillus nidulans*. Mol. Microbiol..

[15] Conlon H., Zadra I., Haas H., Arst H.N., Jr Jones M.G., Caddick M.X. (2001). The *Aspergillus nidulans* GATA transcription factor gene *AreB* encodes at least three proteins and features three classes of mutation. Mol. Microbiol..

[16] Michielse C.B., Pfannmuller A., Macios M., Rengers P., Dzikowska A., Tudzynski B. (2014). The interplay between the GATA transcription factors AreA, the global nitrogen regulator and AreB in *Fusarium fujikuroi*. Mol. Microbiol..

[17] Shi Y.J., Shi Y. (2004). Metabolic enzymes and coenzymes in transcription—A direct link between metabolism and transcription?. Trends. Genet..

[18] Chaudhry M.T., Chaudhry R. (2019). Molecular modeling and in silico characterization of nitrogen metabolite repressor NmrA of opportunistic human pathogen *Aspergillus fumigatus*. J. Microbiol. Biotech. Food. Sci..

[19] Huberman L.B., Wu V.W., Kowbel D.J., Lee J., Daum C., Grigoriev I.V., O’Malley R.C., Glass N.L. (2021). DNA affinity purification sequencing and transcriptional profiling reveal new aspects of nitrogen regulation in a filamentous fungus. Proc. Natl. Acad. Sci. USA.

[20] Schönig B., Brown D.W., Oeser B., Tudzynski B. (2008). Cross-species hybridization with *Fusarium verticillioides* microarrays reveals new insights into *Fusarium fujikuroi* nitrogenre gelation and the role of AreA and NMR. Eukaryot. Cell.

[21] Wagner D., Schmeinck A., Mos M., Morozov I.Y., Caddick M.X., Tudzynski B. (2010). The bZIP transcription factor MeaB mediates nitrogen metabolite repression at specific loci. Eukaryot. Cell.

[22] Wong K.H., Hynes M.J., Todd R.B., Davis M.A. (2007). Transcriptional control of *nmrA* by the bZIP transcription factor MeaB reveals a new level of nitrogen regulation in *Aspergillus nidulans*. Mol. Microbiol..

[23] Han X., Qiu M., Wang B., Yin W.B., Nie X.Y., Qin Q.P., Ren S.L., Yang K.L., Zhang F., Zhuang Z.H. (2016). Functional analysis of the nitrogen metabolite repression regulator gene *nmrA* in *Aspergillus flavus*. Front. Microbiol..

[24] Fernandez J., Wright J.D., Hartline D., Quispe C.F., Madayiputhiya N., Wilson R.A. (2012). Principles of carbon catabolite repression in the rice blast fungus: Tps1, Nmr1-3, and a MATE-family pump regulate glucose metabolism during infection. PLoS Genet..

[25] Macios M., Caddick M.X., Weglenski P., Scazzocchio C., Dzikowska A. (2012). The GATA factors AREA and AREB together with the co-repressor NMRA, negatively regulate arginine catabolism in *Aspergillus nidulans* in response to nitrogen and carbon source. Fungal. Genet. Biol..

[26] Du Y., Jin K., Xia Y. (2018). Involvement of MaSom1, a downstream transcriptional factor of cAMP/PKA pathway, in conidial yield, stress tolerances, and virulence in *Metarhizium acridum*. Appl. Microbiol. Biot..

[27] Liu J., Cao Y.Q., Xia Y.X. (2010). *Mmc*, a gene involved in microcycle conidiation of the entomopathogenic fungus *Metarhizium anisopliae*. J. Invertebr. Pathol..

[28] Zhang S.Z., Peng G.X., Xia Y.X. (2010). Microcycle conidiation and the conidial properties in the entomopathogenic fungus *Metarhizium acridum* on agar medium. Biocontrol. Sci. Technol..

[29] Peng G.X., Xia Y.X. (2011). The mechanism of themycoinsecticide diluent on the efficacy of the oil formulation of insecticidal fungus. BioControl.

[30] Guo H.Y., Wang H.J., Keyhani N.O., Xia Y.X., Peng G.X. (2020). Disruption of an adenylate-forming reductase required for conidiation, increases virulence of the insect pathogenic fungus *Metarhizium acridum* by enhancing cuticle invasion. Pest. Manag. Sci..

[31] Livak K.J., Schmittgen T.D. (2001). Analysis of relative gene expression data using real-time quantitative PCR and the 2^−ΔΔCt^ method. Methods.

[32] Wang C., Wang S. (2017). Insect pathogenic fungi: Genomics, molecular interactions, and genetic improvements. Annu. Rev. Entomol..

[33] Becker A., Schlöder P., Steele J.E., Wegener G. (1996). The regulation of trehalose metabolism in insects. Experientia.

[34] Elbein A.D., Pan Y.T., Pastuszak I., Carroll D. (2003). New insights on trehalose: A multifunctional molecule. Glycobiology.

[35] Wilson R.A., Gibson R.P., Quispe C.F., Littlechild J.A., Talbot N.J. (2010). An NADPH-dependent genetic switch regulates plant infection by the rice blast fungus. Proc. Natl. Acad. Sci. USA.

[36] Platt A., Langdon T., Arst H.N., Kirk D., Tollervey D., Sanchez J.M.M., Caddick M.X. (1996). Nitrogen metabolite signalling involves the C-terminus and the GATA domain of the *Aspergillus* transcription factor AREA and the 3′ untranslated region of its mRNA. EMBO J..

[37] Kotaka M., Johnson C., Lamb H.K., Hawkins A.R., Ren J., Stammers D.K. (2008). Structural analysis of the recognition of the negative regulator NmrA and DNA by the zinc finger from the GATA-type transcription factor AreA. J. Mol. Biol..

[38] Jackson M.A., Heale J.B., Hall R.A. (1985). Traits associated with virulence to the aphid *Macrosiphoniella sanborni* in 18 isolates of *Verticillium lecanii*. Ann. Appl. Biol..

[39] Lane B.S., Trinci A.P.J., Gillespie A.T. (1991). Influence of cultural conditions on virulence of conidia and blastospores of *Beauveria bassiana* to the green leafhopper, *Nephotettix virescens*. Mycol. Res..

[40] Chandler D., Heale J.B., Gillespie A.T. (1993). Germination of the entomopathogenic fungus *Verticillium lecanii* on scales of the glasshouse whitefly *Trialeurodes vaporariorum*. Biocontrol Sci. Technol..

[41] Altre J.A., Vandenberg J.D., Cantone F.A. (1999). Pathogenicity of *Paecilomyces fumosoroseus* isolates to diamondback moth, *Plutella xylostella*: Correllation with spore size, germination speed, and attachment to cuticle. J. Invertebr. Pathol..

[42] Ortiz-Urquiza A., Luo Z., Keyhani N.O. (2015). Improving mycoinsecticides for insect biological control. Appl. Microbiol. Biot..

[43] Lovett B., St Leger R.J. (2015). Stress is the rule rather than the exception for *Metarhizium*. Curr. Genet..

[44] Chaurasia A., Lone Y., Wani O., Gupta U.S. (2016). Effect of certain entomopathogenic fungi on oxidative stress and mortality of *Periplaneta americana*. Pestic. Biochem. Phys..

[45] Shi C., Chen X.R., Liu Z.J., Meng R.Z., Zhao X.C., Liu Z.H., Guo N. (2017). Oleuropein protects L-02 cells against H_2_O_2_-induced oxidative stress by increasing *SOD1*, *GPx1* and *CAT* expression. Biomed. Pharmacother..

[46] Pichersky E., Gang D.R. (2000). Genetics and biochemistry of secondary metabolites in plants: An evolutionary perspective. Trends. Plant. Sci..

[47] Ugine T.A., Wraight S.P., Brownbridge M., Sanderson J.P. (2005). Development of a novel bioassay for estimation of median lethal concentrations (LC_50_) and doses (LD_50_) of the entomopathogenic fungus *Beauveria bassiana*, against western flower thrips, *Frankliniella occidentalis*. J. Invertebr. Pathol..

[48] Wang F., Sethiya P., Hu X., Guo S., Chen Y., Li A., Tan K., Wong K.H. (2021). Transcription in fungal conidia before dormancy produces phenotypically variable conidia that maximize survival in different environments. Nat. Microbiol..

[49] Vega F.E., Jackson M.A., Mercadier G., Poprawski T.J. (2003). The impact of nutrition on spore yields for various fungal entomopathogens in liquid culture. World J. Microb. Biot..

[50] St Leger R.J., Butt T.M., Goettel M.S., Staples R.C., Roberts D.W. (1989). Production in vitro of appressoria by the entomopathogenic fungus *Metarhizium anisopliae*. Exp. Mycol..

