# Supplementary material for: MaNmrA, a Negative Transcription Regulator in Nitrogen Catabolite Repression Pathway, Contributes to Nutrient Utilization, Stress Resistance, and Virulence in Entomopathogenic Fungus Metarhizium acridum
Source: Biology (Basel). 2021 Nov 12;10(11):1167. doi: 10.3390/biology10111167 (PMC8615229; doi:10.3390/biology10111167)
Supplement: Supplementary file 1 [file biology-10-01167-s001.zip › biology-1457770-supplementary.pdf]

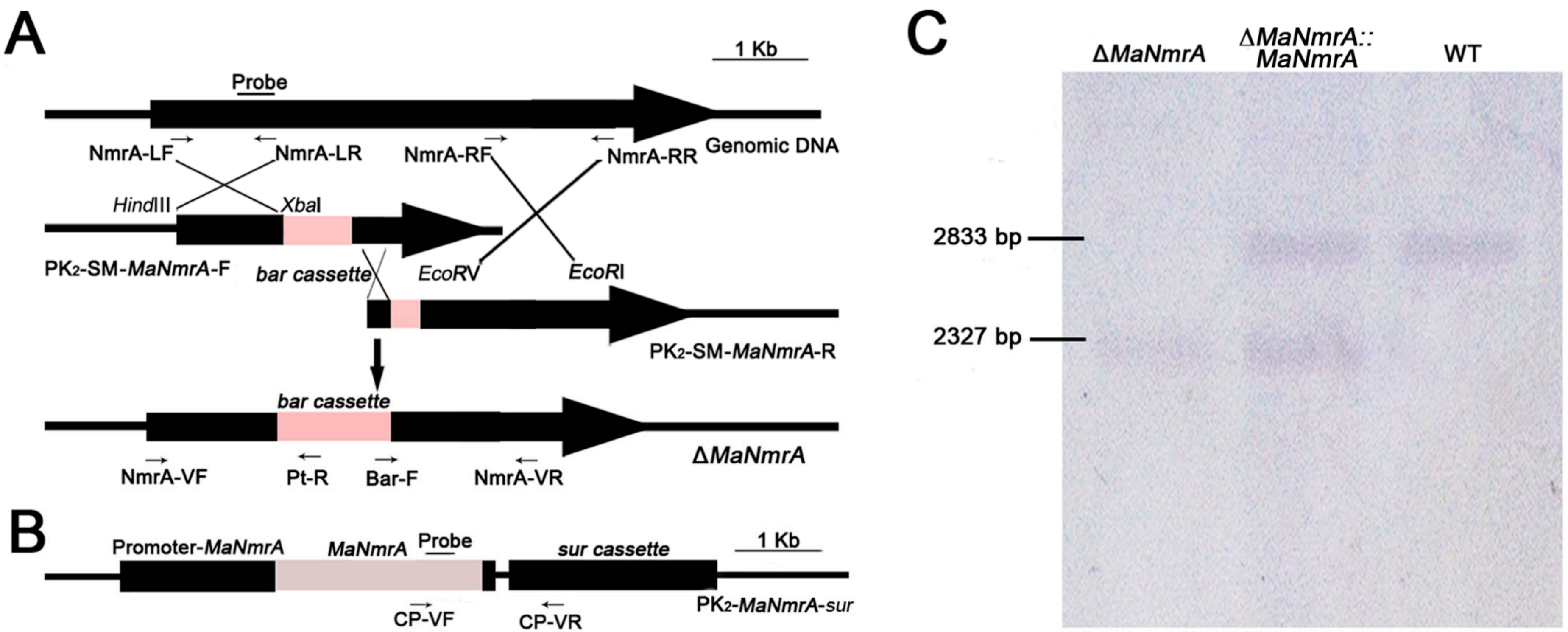

Figure S1 Vector construction and Southern blotting verification. Schematic diagram of *MaNmrA* knockout (**A**) and complemented (**B**) vector constructions. (**C**) Verification of the WT, Δ*MaNmrA* and Δ*MaNmrA::MaNmrA* strains by Southern blotting. Probe was amplified with primers NmrA-PF/NmrA-PR (Table S1). Restriction enzyme *APaI* was used to digest the gDNA of the fungal strains.

Table S1 Primers used in this study

Primer	Sequence (5'-3')	Description
Bar-F	GCTCTACACCCACCTGCT	Universal primer of knockout vector
Pt-R	CAGCCAAGCCCAAAAAGTG	
NmrA-LF	GACGGCCAGTGCCAAGCTGCCACAGCGGTCAGAGTA	For *MaNmrA* knockout vector construction and transformants verification.LF/LR, RF/RR were used for PCR amplification the left and right border sequences, respectively. VF/Pt-R, Bar-F/VR were used for PCR verification the left and right border, respectively.
NmrA-LR	CGGATCCCTCGAGTCTAGTGCGAGGACAGGAAATGC	
NmrA-RF	GCTGGCCGCCCATGGGATGAAATGGAAGGGTGAGCG	
NmrA-RR	ATGACATGATTACGAATTGTCGTCGTCGGAAGTTTG	
NmrA-VF	CACTGATAAGCCTGTGGACTG	
NmrA-VR	GGAACTACGCTGTTGTGGC	
CP-F	GACGGCCAGTGCCAAGCTCGGCGACCTGTTCATCTTAT	For complementation vector construction (CP-F/R) and transformants verification (CP-VF/VR)
CP-R	CCTTGCTCACCATGGATCCTGCCAGCCATTGTTCTTCGT	
CP-VF	ATTGGCATGGCTCTAGCTGATGC	
CP-VR	CGATGCGGTTCACCAGGGTGT	
NmrA-PF	CTTGCTCGGCTTCCTCC	For PCR amplification the probe that used in Southern blotting
NmrA-PR	GGTTTGGTTCGTGGTTGG	
MaMad1-qF	ACATTGTTACCGTTACTATC	Primers used for qRT-PCR
MaMad1-qR	GAATGACAGTCGTAGGAA	
MaMad2-qF	GCACTATGTCCATCCTTG	
MaMad2-qR	TGATGAGGGTCTTGATTTG	
MaPr1-qF	CGGCAAGTATATTGTCAAG	
MaPr1-qR	GTCCTTCTCAATGAAATCG	
MaChit1-qF	TGGCTTCAACTATGGATAT	
MaChit1-qR	GCAGTCTTGTGGTGATAT	
MaGPD1-qF	CACTGAATACGCCGCCTACAT	
MaGPD1-qR	ACTTGACCTTCTTGCCGTTGAC	

Table S1 (continued)

MaNTH1-qF	TGTTGGCTGGCACTGAGAA	Primers used for qRT-PCR
MaNTH1-qR	ACCAGTCCAAGCAAGCATCT	
gpdh-qF	GACTGCCCGCATTGAGAAG	Primers of reference gene used for qRT-PCR
gpdh-qR	AGATGGAGGAGTGGGTGTTG	
ITS-F	TGGCATCTTCTGAGTGGTG	For analysis fungal genomic DNA concentration by absolute quantification
ITS-R	CCCGTTGCGAGTGAGTTA	
